# Indapamide-induced transient myopia with supraciliary effusion: case report

**DOI:** 10.1186/1471-2415-13-58

**Published:** 2013-10-19

**Authors:** Mihály Végh, András Hári-Kovács, Kata Réz, Beáta Tapasztó, Ágnes Szabó, Andrea Facskó

**Affiliations:** 1 School of Optometry, Department of Clinical Ophthalmology, Faculty of Health Sciences, Semmelweis University, 17 Vas utca, Budapest 1088, Hungary; 2 Department of Ophthalmology, Faculty of General Medicine, University of Szeged, 10-11 Korányi fasor, Szeged 6720, Hungary

**Keywords:** Indapamide, Transient myopia, Supraciliary effusion, Ultrasound biomicroscopy

## Abstract

**Background:**

Ingestion of sulphonamide-derived drugs has been reported to possibly have ocular side-effects. Authors aimed to present a rare case of indapamide-induced transient myopia with ciliary body edema and supraciliary effusion.

**Case presentation:**

A 39 years old caucasian female patient presented at the Department of Neurology with headache and sudden bilateral loss of distant vision. Neurological assessment and cranial CT scans were unremarkable. For her hypertension, twice a day bisoprolol 2.5 mg and once a day indapamide 1.5 mg tablets were prescribed several days before. At her presenting, ophthalmic findings were as follows: visual acuity 0.08-7.25Dsph = 1.0 and 0.06-7.25Dsph = 1.0; IOP 25 mmHg and 24 mmHg, anterior chamber depth (ACD) 2.32 mm and 2.49 mm, lens thickness (L) 4.02 mm and 4.09 mm in the right and the left eye, respectively. By means of ultrasound biomicroscopy (UBM), thickened (720 / 700 micron) and detached ciliary body, its forward movement (ciliary body-cornea angle 108′ / 114′) and forward rotated ciliary processes were seen. Angle opening distance (AOD500) were 300 / 314 microns. By the following days, the myopia gradually diminished, and a week after her first symptoms, her uncorrected visual acuity was 1.0 in both eyes, IOP 13 mmHg and 17 mmHg, ACD 3.68 mm and 3.66 mm, L 3.78 mm and 3.81 mm in the right and the left eye, respectively. Ciliary body edema and detachment disappeared (ciliary body thickness 225 / 230 micron), both of the ciliary body-cornea angle 134′ / 140′ and the AOD500 (650 / 640 microns) increased. At this point, the patient admitted that she had stopped taking indapamide two days before.

**Conclusions:**

Our case report is the third one in the literature to present indapamide-induced transient myopia, and the first to employ UBM for describing the characteristics of this rare condition. According to the findings, authors suggest that both ciliary muscle contraction and ciliary body edema may play role in the pathomechanism. UBM seems to be a useful tool in the differential diagnosis of acute myopia. Further, authors wish to draw attention to one of the potential adverse effects of this drug which was not listed by its package insert.

## Background

Several drugs, especially sulphonamide-derived medications which are widely used as diuretics, antibiotics, chemoterapeutics, anti-diabetics, anti-hypertensives as well as synthetic hormones are widely recognised to cause both functional and morphological changes in the ciliary body, finally resulting in acut myopia [1]. There are theories [2,3], but the pathophysiology of the sulphonamide induced myopia has not been discovered yet.

In this study, a case of bilateral acute myopia followed by ingestion of indapamide is reported. It represents the third publication of indapamide-induced pseudomyopia and the first one on its morphological characteristics described by means of high frequency ultrasound biomicroscopy (UBM).

## Case presentation

**Figure 1:**
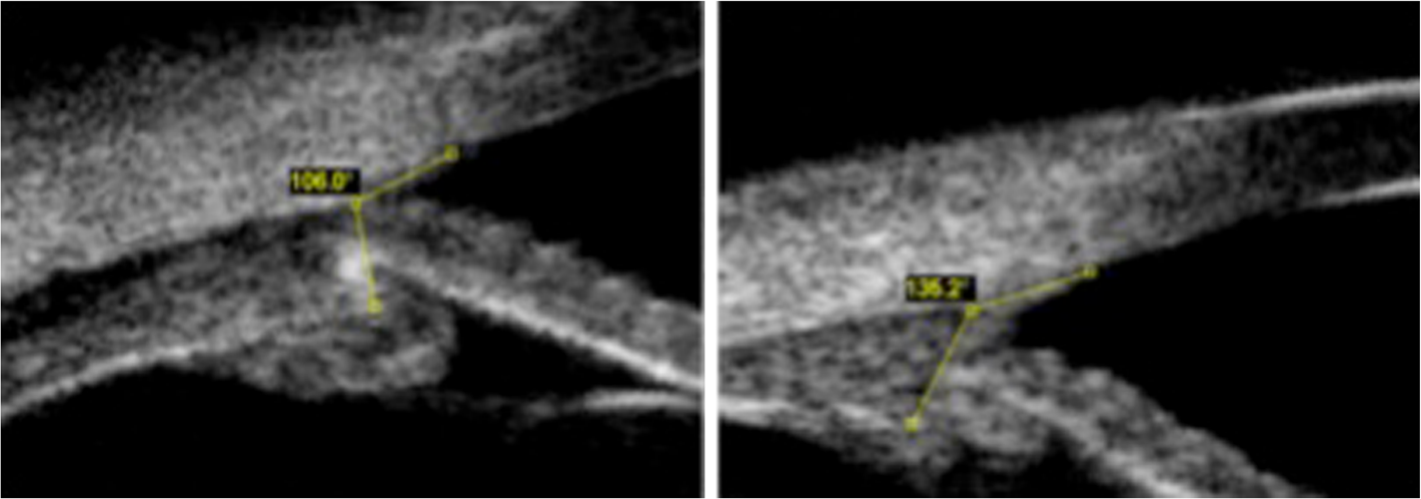
**Ciliary body-cornea angles during the acute phase (left side) and the convalescence (right side).** The angle between the anterior aspect of the ciliary body and the inner surface of the cornea reflects the forward movement of the ciliary body. Several days after discontinuation of taking indapamide, the ciliary body-cornea angle increased by approximately 25 degrees compared to the acute phase (left side).

**Figure 2:**
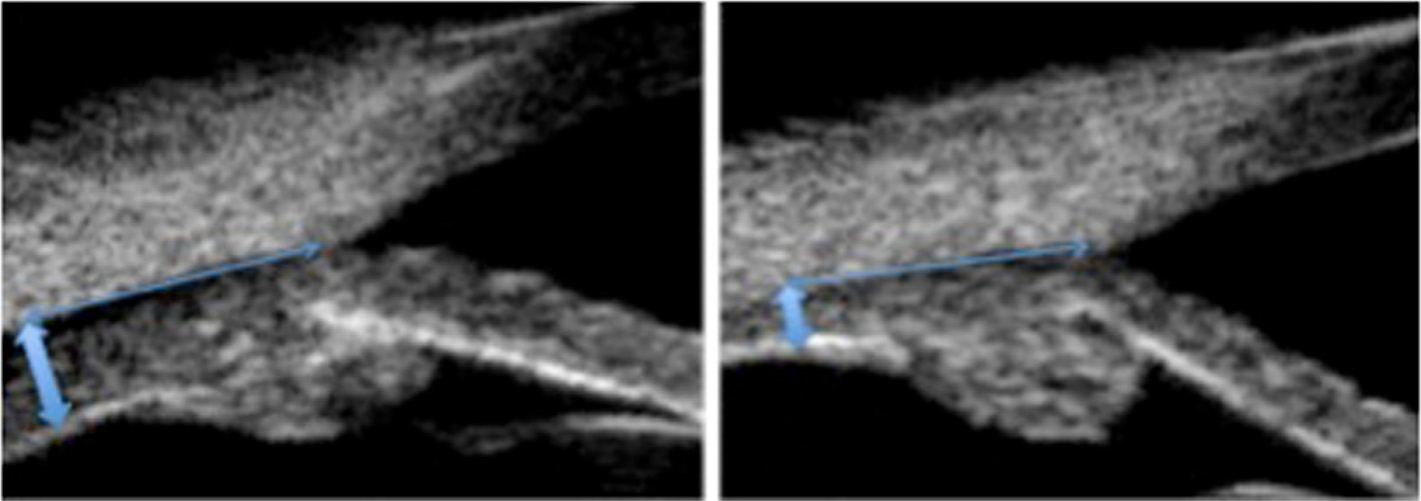
**Ciliary body thickness during the acute fase phase and the convalescence.** The ciliary body thickness (thick double arrows), measured at a 2.0 mm distance (thin double arrows) from the scleral spure, was doubled during the acute phase (left side) compared to the normal value (right side).

**Figure 3:**
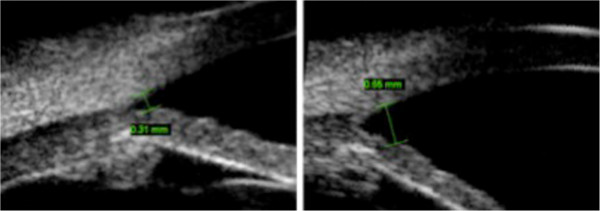
**Changing of angle opening distance.** The angle opening distance measured between the inner aspect of the cornea and the anterior surface of the iris at a 500 microns distance from the scleral spure. During the acute phase (left side), it was markedly decreased compared to that of convalescence (right side).

A 39 years old female patient was referred to our out-patient clinic for consultation by the Department of Neurology due to headache and sudden bilateral visual impairment. Neurological assessment and cranial CT, MRI scans and cerebrospinal fluid samples were unremarkable. In her short time general history, an increased psychic stress followed by hypertension maybe of importance. For that, twice a day medazepam 10 mg, twice a day bisoprolol 2.5 mg and once a day indapamide 1.5 mg tablets were prescribed. Neither of the drugs’ package insert listed myopia as potential side-effect. In particular, she was complaining about the loss of distant vision and having blunt pain in the outer corners of the eyes on attempted reading while the near vision was preserved. On her general ophthalmic evaluation, Snellen visual acuity was 0.08-6.0Dsph = 1.0 and 0.06-6.0Dsph = 1.0 and intraocular pressure measured 25 mmHg and 24 mmHg in the right and the left eye, respectively. Normal versions and convergence, slight esophoria for near and orthophoria for the distance were seen. Mid-dilated, equal pupils with normal direct and consensual light reflexes, shallower anterior chamber, clear medias, and unremarkable fundi could be observed. On gonioscopy, narrow and moderately open parts (Shaffer Grade 1, 2) of the angle could be seen. The confrontation visual field test was normal for both eyes. After instilling a drop of cyclopentolate hydrochloride 0.5% into both eyes, the refractive error decreased to-5.0D in both eyes. According to the above findings, accommodative spasm resulting in (pseudo) myopia was diagnosed. The next day, her headache diminished, other complaints were substantially unchanged. The visual acuity was 0.08-7.0Dsph = 1.0 / 0.06-7.0Dsph = 1.0; IOP 24 / 26 mmHg. Optical biometry revealed shallower anterior chamber but normal lens thickness in both eyes. To obtain more information about the morphological background of the acute myopia, ultrasound biomicroscopy (VuMax 35 MHz, Sonomed Escalon Inc., NY, USA) was employed. The measurements were performed in four quadrants of each eye and the average of the values was taken into consideration for comparing the examinations of different visits. The ciliary body, throughout its “visible” extent was markedly thickened, the suprachoroidal space showed a very low reflectivity with a few more reflective membrans suggesting ciliary body-choroidal detachment and effusion. ‘Insert Figure 1 here’. The anterior aspect of the ciliary body moved forward which was expressed by the ciliary body-cornea angle (CBCA). ‘Insert Figure 2 here’. The ciliary processes rotated remarkably forward, occasionally attaching the posterior aspect of the iris. Angle opening distance was markedly decreased. ‘Insert Figure 3 here’. After putting 3-3 drops of cyclopentolate hydrochloride 0.5% into both eyes, with 15 minutes intervals, the refraction measured-0.75 /−0.50Dsph. Two days later, at her next follow-up appointmet, the patient denied the symptoms she had been complaining about. In that morning, she woke up with clear distant vision and without headache or discomfort around her eyes. The uncorrected visual acuity was again 1.0 / 1.0, IOP 13 / 17 mmHg, the anterior chamber deep and clean. Ciliary body edema and detachment disappeared, both of CBCA and AOD500 increased (Table 1). The patient admitted that she had discontinued taking indapamide two days before on her own, thinking her blood pressure lowered too much.

**Table 1:** Biometric data of the anterior segments of the right/left eyes during acute phase and convalescence

	**Acute phase**	**Convalescence**
**ACD**	2.32 / 2.49 mm	3.68 / 3.66 mm
**L**	4.02 / 4.09 mm	3.78 / 3.81 mm
**AOD500**	300 / 314 micron	630 / 644 micron
**CBT2**	720 / 700 micron	340 / 350 micron
**CBCA**	108 / 114°	140 / 134°

ACD = anterior chamber depth, L = lens thickness, AOD500 = angle opening distance, CBT2 = ciliary body thickness, CBCA = ciliary body-cornea angle.

Since then, she was examined twice and had a full vision in both eyes. However, she has been complaining about mild difficulties in focusing for different distances.

## Conclusions

In the literature, there are only two articles on indapamide caused transient myopia [4,5]. To explain the exact machanism of drug-induced refractive changes three potential contributors have been suggested, namely the osmotic disturbancies of the lens leading to thickening and changing its refractive index; then the ciliary body edema and finally, the accomodative spasm of the ciliary muscles. Both of these can result in the anterior displacement of the iris-lens diaphragm with decreased anterior chamber depth and narrowing of the irido-corneal angle, occasionally with angle closure glaucoma [6-9]. Among the four publications [3,7-9] employing UBM to investigate the related morphological changes, there seems to be a consensus about the role of the lens in the pathomechanism. Although, some increase of the lens thickness can always be observed, the thickening does not correlate with either the anterior chamber shallowing or the myopic shift. Additionally, the transient myopia and angle closure glaucoma can be developed by pseudophakic patients, too [6]. In our case, the lens is unlikely to have a significant role as its average thickening was 0.2-0.3 mm while the anterior chamber flattening was five times more, around 1.1-1.2 mm.

Ciliary body engorgement and supraciliary effusion are likely resulted from the excessive ciliary body edema. Krieg and co-workers analyzing two cases of drug-induced myopia proposed a new theory on the ciliary body swelling. In the first case, the symptoms presented during pregnancy after ingestion of chlorthalidone, in the second one, acetazolamide administration resulted in myopia with marked spastic component in a patient with aspirin-sensitive asthma. Taking into consideration, that both drugs are proved to stimulate the synthesis of prostaglandins just like pregnancy does, and that the levels of spasmogenic leucotriens are higher in asthma, they concluded that edema could be traced to a disturbance in eicosanoid metabolism. The prostaglandins might be responsible for the miosis and, through the vasodilatation and increased permeability of the capillaries, for the edema; while leucotriens for the ciliary body spasm.

Ramos-Esteban at al. stated that the spasm of accomodation is the most unlikely mechanism in the sulfonamide caused transient myopia as instillation of cycloplegic drops almost never abolishes the refractive change [3]. Our case does not support the above statement since the patient had a marked discomfort during close work indicating a spasmic component over and above, cyclopentolate significantly improved her pain as well as the refractive errors. Furthermore, exclusively in this study, the drug-induced forward movement of the ciliary body was measured. It is known from animal studies [10] that the forward movement of the ciliary body achieved by the contractions of the longitudinal parts of the ciliary muscle plays a paramount role in the physiologic process of accomodation. We have found a significant forward movement reflected by an average of 30° lessening in CBCA suggesting that the accomodative spasm of the ciliary muscle, additionally to its edema, also contributed to the development of the symptoms.

The anterior segment changes may give rise to angle closure glaucoma. The therapy consists of prompt discontinuation of taking the causative drug and instilling cycloplegic drops. YAG-iridotomy is of no effect and pilocarpine is contraindicated [2,7,9]. The IOP was slightly raised in our patient at the first two visits but the angle remained open, and the pressure gradually lowered during the following visits as did the refractive error. Probably, the shorter exposure to the medication (she took the indapamide just for 3 days) and the relatively bigger eyes (axial length was 24.06 and 24.02 mm in the rigtht and left eye, respectively) prevented the patients from developing angle closure glaucoma. It is of interest, that neither of the other two cases of indapamide induced acute myopia developed angle closure glaucoma. Beyond the ciliochoroidal detachment, Blain and co-workers noticed diffuse choroidal thickening at the posterior pole and scattered islands of delayed fluorescein filling at the early and midstage fluorescein angiography suggesting transient lobular choriocapillary hypoperfusion [5], presumably, related to choroidal thickening. In our case, the posterior pole was normal and full vision was all the time preserved.

Our case presentation is the third report on indapamide-induced acute myopia and the first to describe the morphological characteristics of this condition by UBM. The co-existence of the centripetal thickening and the forward movement of the ciliary body suggested that both the edema of the ciliary body and the spasm of the ciliary muscles could contribute to the pathogenesis of the myopia caused by indapamide. Such, the therapy, beyond the cessation of the causative drug, should consist of both topical steroids and cycloplegic agents.

## Consent

Written informed consent was obtained from the patient for publication of this case report and all accompanying images. A copy of the written consent is available for review by the Editor of this journal.

## Competing interests

The authors declare that they have neither financial nor non-financial competing interests in relation to this manuscript.

## Authors’ contributions

AF suggested this case report and participated in its development and coordination. ÁS was the main physician responsible for the patient. AHK performed UBM examinations, and was involved in manuscript writing as well as KR. MV and BT helped in reviewing literature sources for this manuscript and helped to draft the manuscript. All authors read and approved the final manuscript.

## Pre-publication history

The pre-publication history for this paper can be accessed here:

http://www.biomedcentral.com/1471-2415/13/58/prepub
